# Laser Micro-Texturing of Sintered Tool Materials Surface

**DOI:** 10.3390/ma12193152

**Published:** 2019-09-26

**Authors:** Daniel Pakuła, Marcin Staszuk, Małgorzata Dziekońska, Pavel Kožmín, Adam Čermák

**Affiliations:** 1 Institute of Engineering Materials and Biomaterials, Silesian University of Technology, Konarskiego Street 18A, 44-100 Gliwice, Poland; 2 HOFMEISTER s.r.o, Mezi Ploty Street 12, 326 00 Pilsen, Czech Republic

**Keywords:** cemented carbides, sialon tool ceramics, laser texturing, Laser Induced Periodic Surface Structures (LIPSS)

## Abstract

The purpose of this paper is to show the effect of tool materials surface treatment while using laser texturing on the structure and properties of cemented carbides and sialon ceramics. The tests were made on multi-point inserts subjected laser texturization and honeycomb-like texture was obtained. Comprehensive investigations in the scanning electron microscope (SEM) were made. Morphology was examined by the use of atomic forces microscope (AFM) and confocal microscope. The chemical composition of the tested materials using energy-dispersive X-ray spectrometer (EDS) was investigated. Moreover, exploitative properties, including wear resistance using the "pin on disc" method and roughness, were also tested. It was found that the laser texturing provides a suitable modification of the structure improving tribological properties. Tests suggest that laser texturing can contribute to the durability of cutting tool’s edge, which qualifies this type of surface treatment for wide industrial applications.

## 1. Introduction

Tool materials that are used on the cutting edges during work are exposed to high loads caused by the resistance of the workpiece, because of these, various methods of improving their cutting properties are used. The improvement of the usable properties of sintered cutting edges for dry cutting at high speeds is possible not only by modifying the chemical composition or changing the edge geometry, but above all by using special surface treatment that consists of manufacturing wear resistant coatings by physical vapor deposition PVD or chemical vapor deposition CVD methods [1,2,3,4]. So far, few attempts have been made to improve the mechanical properties of this material group through the use of laser surface texturing [5,6].

Nowadays, the laser treatment of materials surface is very popular, and it is one of the most avant-garde and effective technology. Laser radiation enables many precise technological operations on various materials due to its properties, with efficiency and accuracy that are far superior to conventional methods. Laser treatment is characterized by non-contact, selectivity, and possibility of full automation. Laser surface texturing is one of the elementary processes of laser micromachining. It consists in a local and short-term thermal or photochemical action specially focused, profiled laser beam on various materials in order to remove, melt, heat up or change their properties. The most important advantages of laser micromachining are: small field of operation, remote operation (non-contact), short-term operation, high process efficiency, and easiness in automation of laser devices [5,6,7,8,9,10,11,12,13,14,15,16,17,18,19,20,21,22,23,24,25,26,27,28,29].

During texturing that is carried out by thermal methods, i.e., laser ablation or electrodial discharge, structural changes take place in the surface layer. One of the objectives of using surface laser texturing in relation to tribology is improving the surface layer material by: hardening or alloying, resulting the possibility to transfer larger loads, and reducing tribological wear [8].

Laser treatment is very often used in modern material engineering, among others for the production of superhydrophobic polymeric surfaces to create hydrophobic polymer areas through laser sculptured microstructure [10]. Another application is the possibility of direct structuring in the areas of thin films of various metals (chromium, aluminum, gold, copper, and silver) deposited on a glass substrate [11]. An interesting application of laser treatment is the creation of cones, at various angles on the surface of silicon, depending on the polarization of the laser beam [12].

Surface texturing is a great option of surface engineering, resulting in significant improvement in wear resistance, friction coefficient of materials exposed on mechanical wear. Various techniques can be employed for surface texturing, but Laser Surface Texturing (LST) is probably the most advanced so far. These methods allow to create a very large number of lubricant micro-reservoirs. These micro-reservoirs can have many functions: can create places where lubricant reserves are accumulated, can create places to which pollutants from the dry friction process are discharged and, in the case of full lubrication, these places can act as a hydrodynamic bearing [21].

Laser texturing results in the formation of so-called LIPSS structure—Laser Induced Periodic Surface Structures—that are dependent on the wavelength used (width of individual ripples) and polarization (LIPSS orientation is perpendicular to the direction of polarization). LIPSS significantly improves the properties of the surface layers of engineering materials. They have found a wide application in photonics, biomedicine, heat transfer, wettability, tribology, and other areas [5,6,10,11,12,13,14,15,16,17,18].

The aim of this work is to examine the effect of laser texturing on the structure and tribological properties of the surface layer of sintered tool materials. The investigations that are presented in this work are preliminary researches. According to the authors’ knowledge, they are one of the first studies of this type on these substrates. Similar studies have been carried out by the authors on a CVD-deposited coating on a sialon ceramic substrate, as described in [5]. 

## 2. Materials

**Figure 1:**
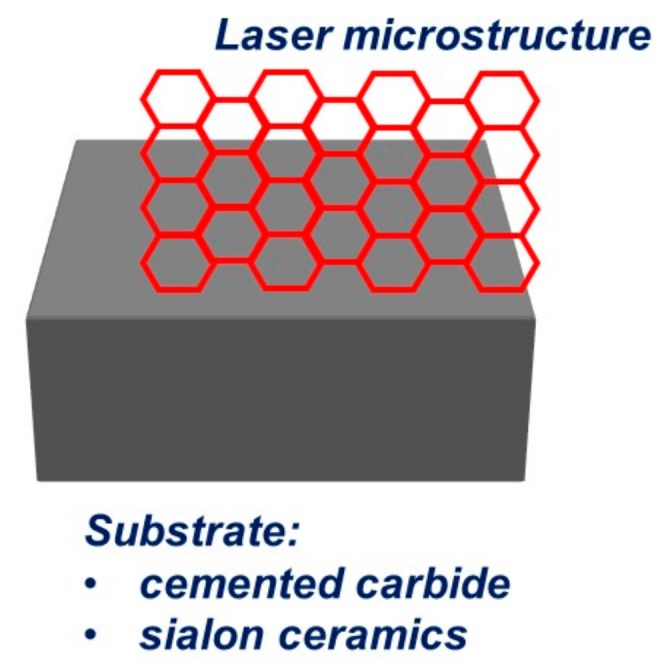
Diagram of laser micro-texturing idea of sintered tool materials surface.

The conception of the presented work is to investigate the influence of laser texturization on the structure and tribological properties of surface sintered tool materials: H10S cemented carbides and sialon ceramics. The tests were made on multi-point inserts that were prepared in accordance with the concept that is shown on Figure 1. There are many types of texture patterns in world literature. [19,20]. A honeycomb-like texture was used in this work.

## 3. Methodology

The laser microstructure was performed while using a pulse laser at Hofmeister in Pilsen. The concept, laser efficiency and geometry of the obtained laser microstructure are protected in Europe by the patents numbered: Patent—no č. 30 072, (2016) in Czech Republic; Patent—no 20 2017 104 373 (2017) in Germany. A neodymium Nd:YVO4 laser was used for texturing the surfaces of tested tool materials—sintered carbides and sialon ceramics. Process parameters were selected from the following ranges possible to obtain for this type of laser:

- λ = 1064 nm–532 nm
- f_p_ = 200–1000 kHz
- P > 20 W (f_p_ = 200 kHz); P > 12 W (f_p_ = 200 kHz)
- τ < 13 ps
- spatial mod TEM00, parameter M^2^ < 1,3
- focusing lens—telecentric
- f (focusing length)—130 mm
- working field of scanning head—45 mm × 45 mm

Scanning electron microscope Zeiss Supra 35 (Jena, Germany) was used to observe both the structure and morphology of the obtained surface layers as well as surface damages resulting from carried out tribological tests. The detection of secondary electrons SE, with acceleration voltage of 5–20 kV and maximum magnification of 60,000 times were used to obtain images of the examined samples. The qualitative and quantitative analysis of chemical composition in micro-areas was made with the Energy Dispersive Spectrometry (EDS) method while using spectrometer EDS LINK ISIS Oxford (Oxford Instruments, Abingdon, UK). 

The LIPSS nanostructure investigations were carried out using the atomic forces microscope (AFM) atomic force microscope Park Systems XE-100 (Suwon, Korea) in a non-contact mode. The obtained results were determined by parameters describing the surface roughness—a Rough Mean Square, (RMS/Rq), the arithmetic average of ordinates profile (Ra), and sum of maximum height and maximum depth (ΔZ). The parameters were calculated over three scan areas 20 µm × 20 µm and 5 µm × 5 µm. The tests were made in a non-contact mode with the use of a silicon measuring beam (cantilever) with constant spring 40 N/m, frequency 300 kHz, blade height about 15 μm, and tip ROC (radii of curvature) about 6 nm.

Surface topography investigations of manufactured textures and wear profiles were also made while using a Zeiss Smartproof5 confocal microscope.

Tribological tests were carried out on the CSM “pin-on-disc” tester (Tribometer CSM Instruments, Anton Paar GmbH, Peseux, Switzerland) under the following conditions: counter-sample-ball made from the Al_2_O_3_ aluminum oxide with 6 mm diameter, counter-sample load 5 N, friction radius 5 mm, linear velocity 0.1 m/s, ambient temperature 20 °C. For all tested samples, the same distance of wear track was assumed—1000 m. 

The surface roughness and abrasion profiles were measured with a Surftec 3+ profilometer Rank Taylor Hobson (Surftec, AMETEK’s Ultra Precision Technologies Group, Berwyn, Pennsylvania, USA).

## 4. Results and Discussion 

**Figure 2:**
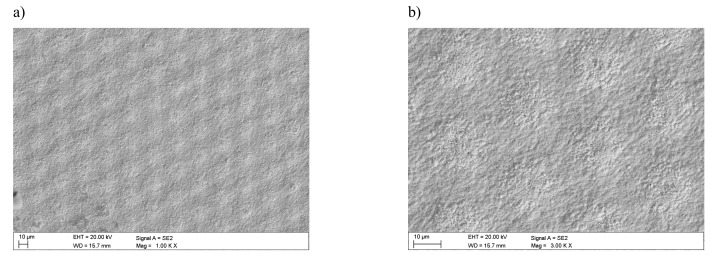
Laser microstructure on cemented carbides: (**a**) area 1000×, (**b**) area 3000×.

**Figure 3:**
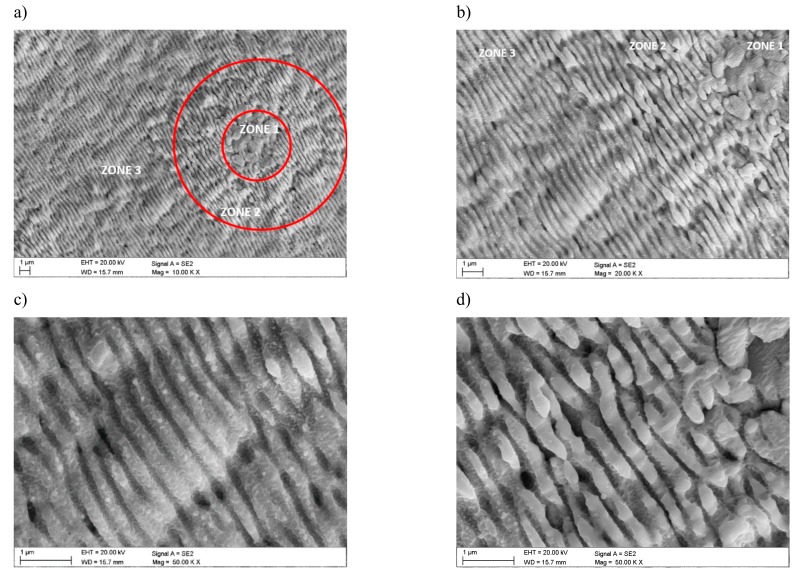

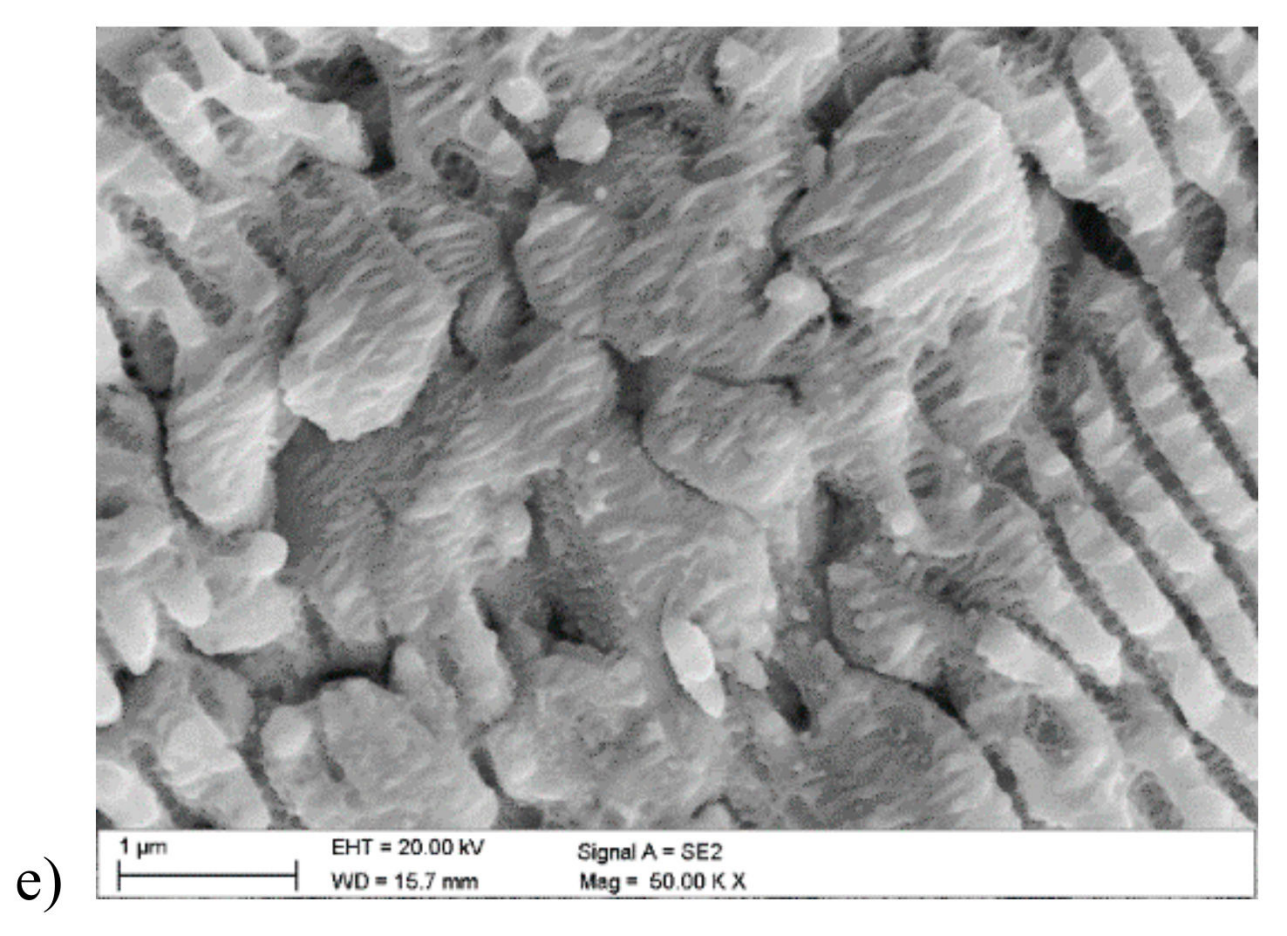
Nanoripples laser-induced periodic surface structures (LIPSS) on surface cemented carbides (**a**) area 10,000×, (**b**) area 20,000×, (**c**) area 50,000×—zone 3, (**d**) area 50,000 x—zone 2, and (**e**) area 50,000 x—zone 1.

Investigations that were conducted in the scanning electron microscope confirmed the formation of a honeycomb-like microstructure and the occurrence of lubrication reservoirs (Figure 2). In addition, detailed studies allow for stating that the selective laser texturing of the surface layer of tested tool materials causes fragmentation of the microstructure within the zone of laser beam impact. They are also marked as laser-induced periodic surface structures (LIPSS), termed nanoripples. Visible crystal texture is characterized by the same shape and similar width of the resulting LIPSS nanoripples. It was also observed that these ripples show orientation towards a certain direction—i.e., axial crystalline texture (Figure 3).

The Figure 3 shows the periodic nanostructures on the cemented carbides surface that was obtained by picosecond (10 ps, wave length 532 nm) laser micromachining. In this Figure, it can be seen three different areas with those nanostructures. Their periodicity is close to the laser wavelength and it depends on many other material and laser properties, which are fluence, number of pulses and number of scans, angle of incidence, wavelength, polarization, and scanning velocity, as can be seen in three areas. 

Laser polarization is one of the most important parameters during the formation of LIPSS, because the morphology (structure) of the pattern that is obtained in the nanoscale and its direction are controlled by laser-pulse electric field.

In the first zone, a melted, irregular structure was observed, on which few and irregular laser-induced periodic surface structures (LIPSS) are arranged perpendicular to the pulse polarization. Accordingly, the created structure of LIPSS is related to the direct effect of the focus position. Moving away from the focus position of the laser, more distinct and regular structure of the LIPSS nanoripples (zone 2 and 3) is formed. In zone 2 were observed clear, sharp edges LIPSS, which were arranged parallel to the pulse polarization on which also appear nanostructures perpendicular to the direction of the laser coming from zone 1. In zone 3, the most far away from the impact of pulse polarization, LIPSS become more blurred.

In zone 2, approaching the surface to the focus position, the fluence is higher, and low-spatial frequency LIPSS (LSFL) with period around the pulse wavelength are formed, while in zone 3 the surface is significantly out of the focus, at low fluence, high-spatial frequency LIPSS (HSFL) with period below the pulse wavelength. Moving further towards the focal position, the local fluence of the central part of the beam exceeds the melting threshold of the material, which was also confirmed in [18] by Gregorcic P., who received similar structures on cold work tool steel.

**Figure 4:**
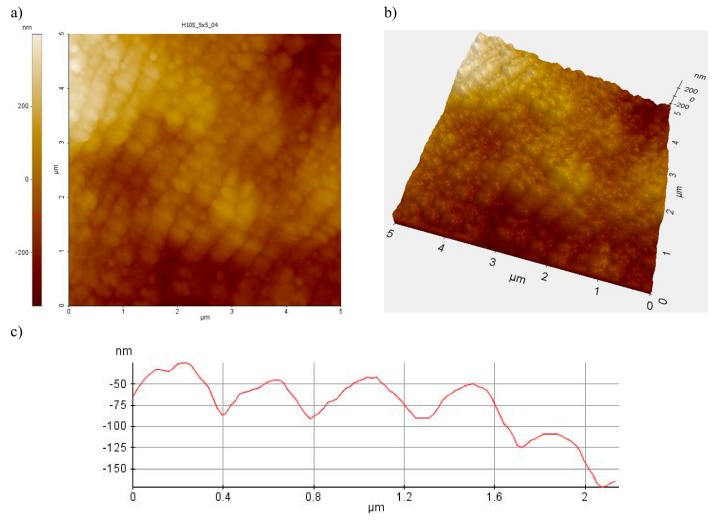
Nanoripples LIPSS (**a**) view two-dimensional (2D)-scan area 5 μm × 5 μm, (**b**) view three-dimensional (3D)-scan area 5 μm × 5 μm, (**c**) profile

The width of the nanoripples formed on cemented carbides in examined zone 2 and 3 varies from 200 to 400 nm, as also confirmed by AFM atomic force microscopy. In turn, the nanoripples height does not exceed 200 nm (Figure 4). A suchlike geometry of the LIPSS structure was also observed by the authors in [5].

**Figure 5:**
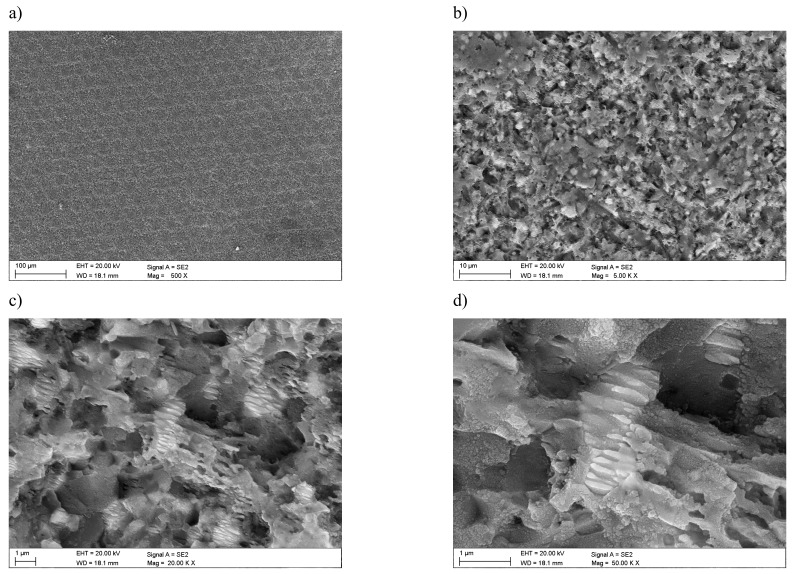
Laser microstructure on sialon tool ceramics: (**a**) area 500×, (**b**) area 5000×, (**c**) area 20,000×, and (**d**) area 50,000×.

A similar fragmented microstructure is visible on the surface of sialon ceramics, although in this case the surface of nanoripples is smaller when compared to the cemented carbides substrate due to the presence of a network of micro pores and micro gaps on this surface (Figure 5). It is worth noting that the structure of ripples is closely related to the surface of the workpiece. Due to the nanometric size, any material flaw, such as scratch, irregularity, or the surface roughness, affects the absorption of the laser beam, and what is connected with it, also the formation of the structure of periodic ripples.

**Figure 6:**
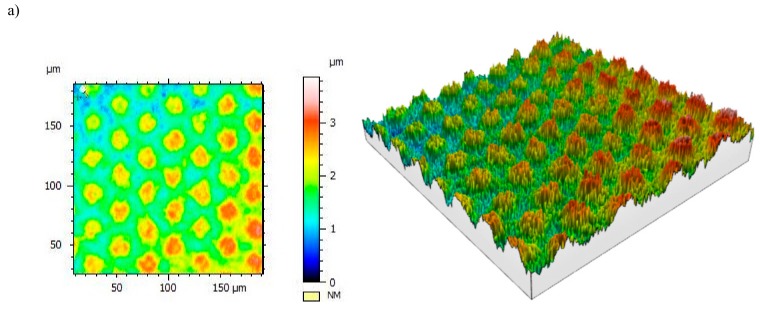

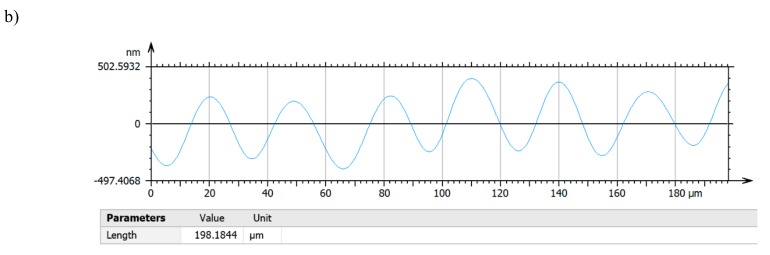
(**a**) Surface topography after laser texturized cemented carbides, (**b**) profile of obtained surface.

Laser confocal microscopy researches confirmed the presence of porous microstructure with a uniform distribution of pores. The pores are evenly positioned about 30 μm apart. Selective laser texturing led to the formation of the so-called micro/nano-lubricating reservoirs (Figure 6).

**Figure 7:**
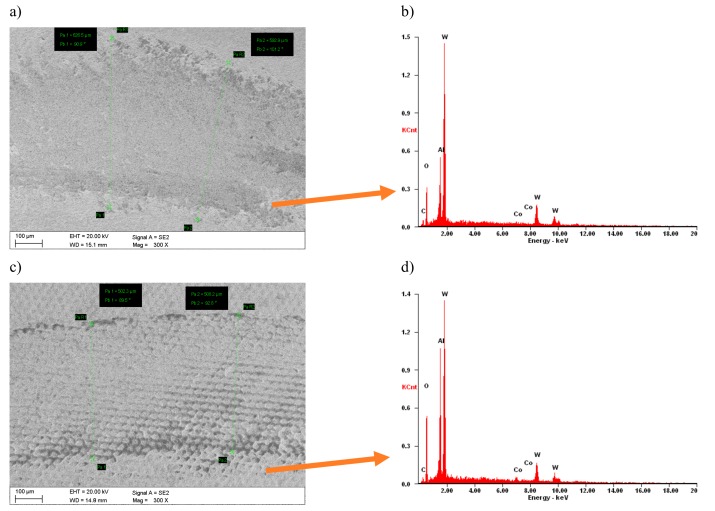
Wear resistance of cemented carbides (**a**,**b**) before laser texturing, (**c**,**d**) after laser texturing.

**Figure 8:**
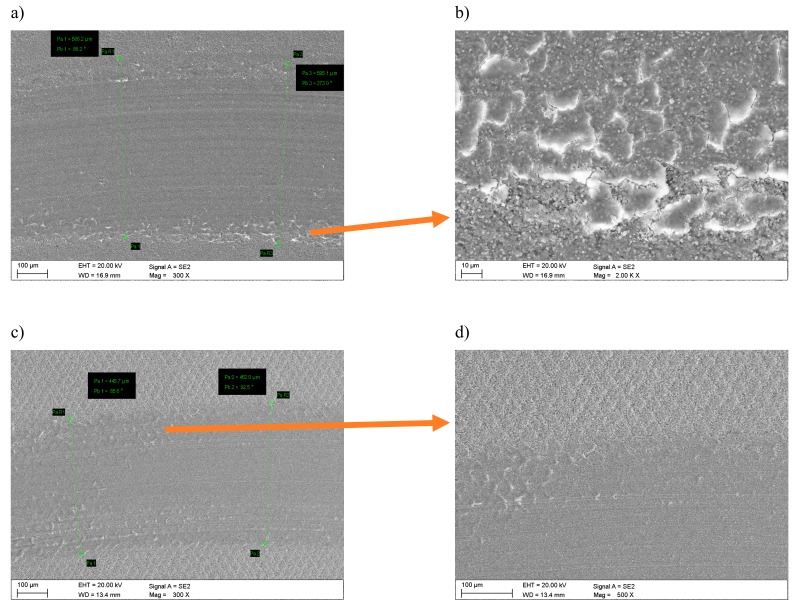
Wear resistance of sialon ceramics (**a**,**b**) before laser texturing, (**c**,**d**) after laser texturing.

**Figure 9:**
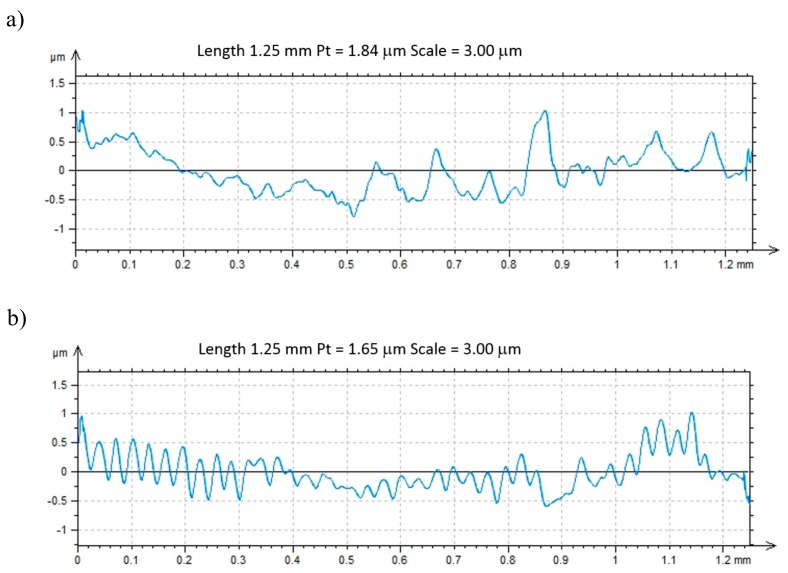
Cross section profiles of cemented carbides wear during pin on disc test: (**a**) before laser texturing, (**b**) after laser texturing.

Tests of abrasion resistance of examined tool materials using the “pin-on-disc” method show that laser texturing improves the tribological properties. The width of wear trace of the cemented carbides before laser texturing is 620 μm, and after laser texturing 500 μm (Figure 7). In turn, the sialon ceramics width of wear trace before laser texturing is 590 µm and 450 µm after laser texturing (Figure 8). For all of the tested materials, the damaged surfaces adhere to the counter-sample. The most counter-sample material adheres to sialon ceramics due to topography of the surface, as confirmed by the point microanalysis of the chemical composition EDS from the investigated area (Figure 7b,d). Wear of tested materials surfaces after laser texturing is more even along edges of the tribological trace. It was also noticed that the micro/nano structure formed reduced the contact surface with the material counter-sample, thus contributing to the reduction of wear depth, as shown by the example of the wear profiles on cemented carbides (Figure 9).

**Figure 10:**
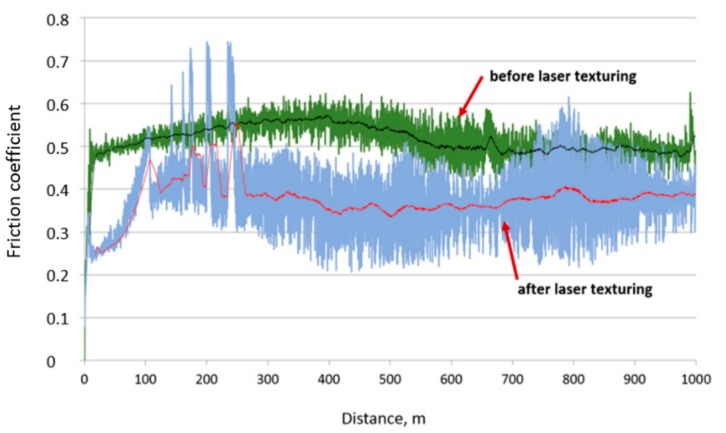
Diagram of friction coefficient according to the friction path during the pin-on-disc test for cemented carbides—before and after laser texturing.

On the base abrasion resistance tests by the "pin on disc" method, it was also found that the value of the friction coefficient after laser texturing decreases. In the case of cemented carbides substrate, the friction coefficient before laser texturing reaches the value of 0.6 for about half of the assumed distance, and then it is about 0.5. On the other hand, after laser texturing at distance 250 m, the friction coefficient value is about 0.35. (Figure 10).

**Figure 11:**
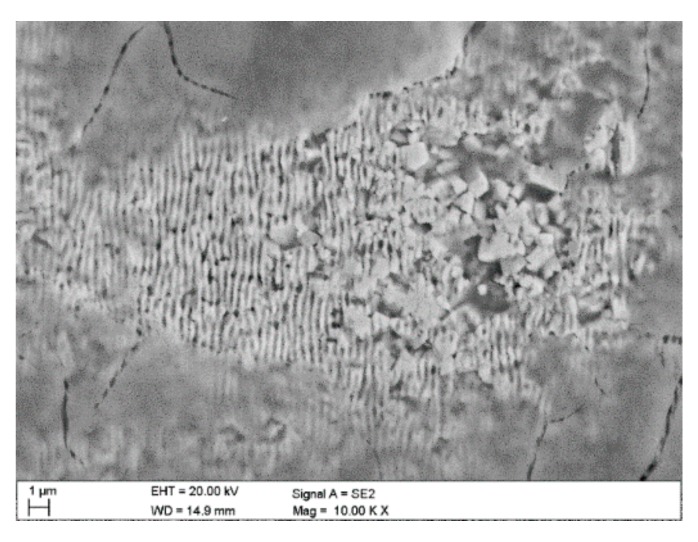
Trace after pin-on-disc of sintered carbides after laser texturing.

During the pin-on-disc test, on textured cemented carbides, it was observed that wear was the highest in the zone 3 was wear, where the tops of nanoripples were abraded and the products of abrasions could accumulate in the cavities, in particular in zone 1 in the so-called lubrication micro/nano reservoirs (Figure 11).

Investigations and simulations that are presented in the work [11] confirm that structures with a shape similar to a circle or a square are the best, and the density of the applied pattern is of great importance—the greater the number of holes, the more regular the distribution of the lubricant on the tool. In investigated materials, the distance between individual holes is approximately 30 μm. This small distance reduces the friction force and lateral forces in the sample—counter-sample system.

In addition, the reduction in the friction coefficient in the studied case was achieved by the formation of LIPSS nanoripples, resembling a shaft with their shape, after which the friction element moved like on rolls, which was also confirmed by the Authors of the work [12].

## 5. Conclusions

The conducted investigations allowed for determining the effect of laser texturing on the structure and tribological properties of the surface layer of sintered tool materials. 

As a result of researches, it was found that sequential laser texturing of the surface layer of tested tool materials—cemented carbides and sialon ceramic tools—causes fragmentation of the microstructure within the zone of laser beam impact. 

The resulting nanoripples LIPSS are regularly placed and they have the same shape and size and are arranged axially along one direction especially in zone 2 and 3. While, in zone 1, the structure is less regular and fewer nanoripples are arranged parallel to each other and perpendicular to LIPSS from zone 2 and 3, which was confirmed by structure tests that were made in scanning electron microscope.

Laser texturing contributes to the formation of lubrication micro reservoirs and, in this case, also nano reservoirs. The resulting micro/nano structure reduces the intensity of wear of the surface of tested tool materials, as observed during the "pin on disc" test. 

It was observed that created lubricant micro-reservoirs are places where impurities accumulate during the abrasion process of the tested materials.

To sum up, it should be noted that the laser texturing of tested sintered tool materials causes a decrease in the width and depth of wear trace and, above all, a significant decrease in the friction coefficient. The carried out abrasion resistance tests suggest that laser texturing can contribute to increasing the durability of cutting tool’s edge, which uniquely qualifies this type of surface treatment, in the future for wide industrial applications.
